# Expectations concerning cancer treatment: perspectives of medical oncologists and patients on advanced, unresectable lung carcinoma

**DOI:** 10.3389/fpsyg.2024.1392567

**Published:** 2024-10-09

**Authors:** Patricia Cruz-Castellanos, Paula Jiménez-Fonseca, Rocío Galán-Moral, Nuria Piera-Molons, Marina Gustems, Caterina Calderon

**Affiliations:** ^1^Department of Medical Oncology, Hospital General Ciudad Real, Ciudad Real, Spain; ^2^Department of Medical Oncology, Hospital Universitario Central de Asturias, Oviedo, Spain; ^3^Department of Medical Oncology, Hospital Universitario Arnau de Vilanova, Lleida, Spain; ^4^Department of Clinical Psychology and Psychobiology, Faculty of Psychology, University of Barcelona, Barcelona, Spain

**Keywords:** cancer treatment, expectation, lung cancer, oncologist, prognosis, toxicity

## Abstract

**Introduction:**

This study seeks to compare expectations regarding systemic cancer treatment for advanced lung cancer from the perspectives of both patient and medical oncologist.

**Methods:**

A cross-sectional study involving 17 medical oncologists from 13 Spanish hospitals between 2021 and 2022. Patients with advanced, unresectable lung cancer were recruited prior to initiating systemic cancer treatment. Both patients and oncologists completed the NEOetic-EIT and the STAR.

**Results:**

Seventeen medical oncologists specializing in lung cancer participated, with a mean age of 36.2 years (range 28–56); 65% were female. The study included 298 patients with advanced, unresectable lung cancer, predominantly non-small cell type (72%), and most at stage IV (77%). Most patients were retired or unemployed (71%), and married or partnered (77%). Treatment approaches varied, with 44% based on biomarkers. Oncologists had greater expectations of positive outcomes for participants with better baseline prognosis, such as ECOG 0, newly diagnosed, locally advanced, unresectable non-small cell lung cancer, and those receiving biomarker-based treatments. In contrast, patients’ treatment expectations did not vary based on sociodemographic or clinical factors. Generally, patients had high expectations of cure, in contrast to oncologists’ lower expectations, though both anticipated similar quality-of-life improvements. Patients anticipated more side effects than oncologists. Among oncologists, expectations varied by gender and decreased with age and experience, with no differences detected among patients based on gender, age, or doctor-patient relationship.

**Conclusion:**

This study reveals the complex expectations of patients and oncologists in advanced lung cancer treatment. It underscores the need for effective communication in oncology to align patient expectations with clinical realities.

## Introduction

Lung cancer remains a significant public health challenge, consistently ranking as one of the leading causes of morbidity and mortality worldwide. Its incidence has seen a steady rise, posing a global health threat. According to the World Health Organization, it was estimated that there were over 2 million new lung cancer cases in 2020, accounting for approximately 11% of all cancer cases (World Health Organization, 2022; Siegel et al., 2020). While the incidence of this disease is increasing, its prevalence remains relatively low due to high mortality rates. With nearly 2.2 million deaths attributed to lung cancer in 2020, it is the leading cause of cancer-related death in both men and women (Alberg and Samet, 2003). Notably, lung cancer treatment has changed significantly in recent years, with the advent of targeted therapies and immunotherapy, drastically improving prognosis.

Expectations regarding cancer treatment in lung cancer encompass a complex interplay of the perceptions and hopes of both patients and oncologists. Such expectations are shaped by a range of factors, from the clinical effectiveness of the treatments to the quality of life experienced during and post-treatment (Calderon et al., 2021). From the patients’ perspective, treatment expectations are closely linked with factors such as the potential for disease cure or control, as well as the side effects and their impact on daily living activities. Open communication between patient and oncologist regarding the probabilities and potential challenges is essential to manage expectations realistically (Berger et al., 2010). On the other hand, for the oncologist, it is crucial to manage treatment expectations to foster effective collaboration with patients and enhance clinical outcomes. From the oncologist’s perspective, expectations often revolve around treatment efficacy, measured by objective parameters such as survival rates, response rates, and quality of life. A balance is typically sought between treatment effectiveness and tolerability, with decisions based on scientific evidence and clinical experience (Abernethy and Grubbs, 2014).

The landmark study on patients’ expectations regarding chemotherapy is Weeks et al. (2012) This study analyzed 1,274 patients with colorectal (*n* = 483) and lung (*n* = 710) cancer in the United States between 2003 and 2005 and found that 69% of patients with advanced, incurable lung cancer receiving chemotherapy did not understand that it was not curative, which could impair their ability to make informed decisions. However, a better understanding of incurability might decrease satisfaction with medical care. This study relied on a single survey conducted 4 months after diagnosis, excluding patients who died early, and the survey was conducted by interviewers, which may have influenced the responses (social desirability bias). In a Japanese cohort of 200 patients with advanced lung cancer treated between 2017 and 2021, 38.5% had unrealistic expectations of a cure (Hasegawa et al., 2024). Although 92% of oncologists reported explaining incurability, only 69% of patients confirmed this. Patients who had repeated discussions about incurability with their oncologist tended to have more accurate prognostic awareness, leading to more informed therapeutic decisions (Hasegawa et al., 2022). Additionally, a 2021 Brazilian study evaluated 90 patients with advanced breast, gynecologic, urologic, or gastrointestinal cancer, and 28 oncologists, finding that although 87.6% of patients wanted information about their prognosis, only 35.2% reported receiving it (Paiva et al., 2022). Furthermore, 61.8% expressed that the information should be conveyed in a way that maintains hope. The agreement between patients and oncologists regarding treatment goals and curability was low. These findings underscore the importance of accurate, continuous, and repeated communication about prognosis and treatment goals to help patients develop realistic expectations.

In a previous study conducted by our group with individuals suffering from advanced, inoperable cancer of various locations, we discovered that oftentimes they appear not to understand that antineoplastic therapy is not curative (Carmona-Bayonas et al., 2023). We found that psychosocial factors such as hope, spirituality, coping based on a fighting spirit, and the diagnosis of cancers other than breast cancer, were linked to an inaccurate prognostic awareness and a belief in cure. These factors proved to be as influential as the information provided by the oncologist. Furthermore, we observed that imprecise prognostic awareness was associated with heightened interest in low-efficacy treatments, while a realistic understanding of prognosis increased anxiety and depression, and diminished quality of life. These findings pose a challenge for the oncologist: to provide accurate information that enables the patient to make informed decisions about treatment, while ensuring that knowledge about realistic prognosis does not cause psychological distress. With all the previous background, this work seeks to compare patients’ and medical oncologists’ expectations about systemic treatment for advanced lung cancer and analyze the factors that contribute to these differences.

## Materials and methods

### Study design and patients

This study was designed as a cross-sectional investigation, conducted in 13 medical oncology departments of Spanish hospitals from January 2021 to December 2022. Adult patients (>18 years) with a recent diagnosis (within the previous month) of histologically confirmed advanced-stage, unresectable non-small cell lung carcinoma (adenocarcinoma or squamous carcinoma) and small cell lung carcinoma were included. Patients with newly diagnosed advanced cancer or with recurrence were recruited, provided that the recurrence occurred at least 6 months after any treatment received for a non-advanced stage. Participants had to be eligible for systemic antineoplastic treatment in the medical oncologist’s opinion. Subjects were excluded if their physical condition, age, or comorbidities contraindicated antineoplastic treatment as per the attending oncologist’s judgment. Individuals who had received treatment for another advanced cancer within the past 2 years or who had underlying medical, sociological, family, or personal conditions that could hinder their participation in the study were also excluded. In each center, one or two medical oncologists responsible for the care of lung cancer patients were designated, selected by the department head, and had at least 6 years of experience in treating these patients.

Patients were recruited consecutively during their first visit to the medical oncologist, where they were informed about their diagnosis, stage of disease, and systemic antineoplastic treatments available. In these routine consultations, the medical oncologist also informed them about the study, ensuring consistent communication across participating sites, and informed consent was obtained prior to inclusion. Participation was voluntary, anonymous, and designed to ensure no interference with the patients’ standard care. The study obtained approval from the Ethics Committee of each participating hospital and the Spanish Agency of Medicines and Health Products (AEMPS; identification code: ES14042015).

### Variables and data collection

Medical oncologists gathered sociodemographic and clinical data through patient interviews, electronic health records, and complementary diagnostic tests, and completed the NEOetic-EIT and STAR-C questionnaires after the initial appointment. Patients filled out the NEOetic-EIT and STAR-P questionnaires at home following their first consultation with the oncologist and prior to commencing systemic antineoplastic therapy. They then gave the questionnaires to the auxiliary study staff at their subsequent healthcare facility visit. The medical oncologists used a web-based platform^1^ with filters and a query system for data collection, regularly reviewed to maintain data integrity and quality.

NEOetic-EIT is a scale that was created, tested, and validated in a previous study with a Spanish sample of patients with advanced cancer (Carmona-Bayonas et al., 2023). It assesses the expectations of both patients and oncologists independently regarding various aspects of systemic cancer therapies, including the likelihood of cure, improvement in quality of life, relief of cancer symptoms, and the presence of significant side effects. Oncologists provide responses based on their own expectations for the treatment’s outcomes, not on what they perceive the patient’s expectations to be.

Responses were rated on a Likert scale, which ranked the probability as unsure, very low, low, high, and very high. Additionally, participants assessed their expectation of the treatment’s efficacy in extending survival beyond 18 months.

The STAR-C (Scale to Assess the Therapeutic Relationship—Clinician version) and STAR-P (Patient version) developed by McGuire-Snieckus et al. (2007), is used to assess the therapeutic relationship from the perspectives of the oncologist and the patient, respectively. Both versions consist of 12 items rated on a 5-point Likert scale (from 0 to 4), allowing each party to evaluate their therapeutic relationship from their perspective. STAR-P includes subscales for assessing positive collaboration, positive clinician input, and non-supportive clinician input, while STAR-C measures positive collaboration, emotional difficulties, and positive clinician input. The original versions have demonstrated acceptable internal consistency, test–retest reliability, and factorial validity, confirming their effectiveness in enhancing and understanding the therapeutic relationship (McGuire-Snieckus et al., 2007).

After 3 months of systemic treatment, patients completed the Common Terminology Criteria for Adverse Events (CTCA-CTC) questionnaire in writing and reported any adverse effects experienced during that period.

### Statistical analysis

Descriptive statistics were utilized to present demographic information for both patients and clinicians, as well as participants’ clinical data. T-test was used to evaluate the expectations of patients and oncologists regarding the outcomes of systemic cancer treatment based on the patients’ sociodemographic and clinical variables. Chi-square test was used to analyze differences in expectations regarding cure, quality of life improvement, and symptom relief between patients and oncologists. Pearson correlation coefficients were computed to explore associations between predictions of treatment efficacy and toxicity, demographic variables, and the therapeutic relationship between patients and clinicians, as assessed through questionnaires. The significance level for statistical analyses was set at 0.05. IBM-SPSS software package for Windows, version 26.0 (SPSS, INC., Chicago, IL, USA) was used for all statistical analyses.

## Results

### Baseline characteristics of the sample

Seventeen medical oncologists from 13 Spanish hospitals and with a high level of specialization in lung cancer participated in the study. Of these, 65% were female with a mean age of 36.2 years (range 28–56), and 11.2 years of experience (range 6–32). No significant differences were detected between male and female oncologists in terms of age (*t* = −1.153, *p* = 0.267) or years of experience (*t* = −1.255, *p* = 0.229); 59% were employed in a public university hospital, 29% in public non-university hospitals, and 12% in private hospitals.

These professionals recruited 312 subjects with advanced, unresectable lung cancer. Fourteen participants were excluded from the study: three for failing to meet inclusion criteria; three for meeting exclusion criteria, and eight for incomplete data. The final patient sample comprised 298 individuals (Table 1). Of these, 37.9% were female with mean age of 65.5 years (range 35–90). Most were married or partnered (77%) and had a primary level of education (40%); 71% were retired or unemployed. The most common performance status was ECOG 0 (43%); 81.2% had more than three Elixhauser comorbidities, the most frequent being arterial hypertension (74%), chronic obstructive pulmonary disease (68%), and diabetes (57%). The cancer characteristics were non-small cell lung cancer (72%), adenocarcinoma (40%). and squamous carcinoma (32%), most were stage IV (76.8%), with the rest being unresectable stages IIIB-C. In terms of treatment modalities, most subjects received combined therapy, either polychemotherapy (36%) or chemotherapy with immunotherapy (37%), while the rest were treated with monotherapy using an immune checkpoint inhibitor (15%) or targeted therapy (12%). Treatment decisions were based on a biomarker in 44% of the cases.

**Table 1:** Patient baseline sociodemographic and clinical characteristics.

Characteristics	*N* = 298	%	Oncologist *p* values	Patients *p* values
Gender (women)	113	37.9	–	–
Age (≥65)	166	55.7	–	–
Marital status (married or partnered)	228	76.5	–	–
Education (primary level)	118	39.5	–	–
Work (retired or unemployed)	212	71.1	–	–
Performance status ECOG (0)	128	43.0	0.001	–
Elixhauser comorbidities (> 3)	242	81.2	–	–
Cancer histology			0.001	–
Non-small cell lung carcinoma	215	72.1		
Small cell carcinoma	83	27.9		
Cancer stage			0.001	–
Locally advanced	69	23.2		
IV	229	76.8		
Cancer biomarker (yes)	132	44.3	0.001	–
*De novo* cancer diagnosis (yes)	258	86.6	0.022	–

### Expectations regarding systemic cancer treatment: patients’ and oncologists’ perspectives based on clinical variables

Medical oncologists believed that patients with higher expectations of good outcomes from systemic cancer treatment were those with non-small cell cancer compared to small cell cancer (*M* = 46.9 vs. *M* = 35.5, *t* = 3.754, *p* = 0.001), subjects with unresectable, locally advanced cancer (stage IIIB-IIIC) vs. those with metastasis (stage IV) (*M* = 58.3 vs. *M* = 39.4, *t* = 6.055, *p* = 0.001), individuals with treatment selected via a biomarker vs. those without (*M* = 49.6 vs. *M* = 39.1, *t* = −3.810, *p* = 0.001), patients with an ECOG performance status of 0 vs. ≥1 (*M* = 50.1 vs. *M* = 38.9, *t* = 4.087, *p* = 0.001), participants with a *de novo* cancer diagnosis vs. those with recurrence (*M* = 51.8 vs. *M* = 42.5, *t* = 2.298, *p* = 0.022), and those with a life expectancy given baseline cancer characteristics of more than 18 months vs. those having a shorter baseline expectancy (*M* = 49.0 vs. *M* = 37.2, *t* = 4.357, *p* = 0.001) (Table 1).

In contrast, no significant differences were found concerning patients’ expectations for good treatment outcomes according to the sociodemographic and clinical variables considered by the medical oncologists. Therefore, they were unable to discern the prognostic impact of their cancer’s baseline characteristics.

When comparing the expectation of survival beyond 18 months with systemic cancer treatment according to tumor histology, significant differences were found in favor of non-small cell carcinoma among oncologists (*M* = 46.9 ± 24.9 vs. *M* = 35.5 ± 19.5, *t* = 3.754, *p* < 0.001), while no significant differences were observed between patients with non-small cell and small cell carcinoma (*M* = 81.4 ± 30.1 vs. *M* = 78.1 ± 32.1, *t* = 0.811, *p* = 0.418).

### Expectations concerning systemic cancer treatment: comparing patients’ and oncologists’ perspectives based on expected outcomes

Both oncologists’ and participants’ NEOetic-EIT questionnaire scores regarding outcome expectations for systemic cancer treatment are presented in Table 2. Expectations of cure from treatment was high among patients while it was low or very low among oncologists (75 and 10%, respectively). Both subjects and physicians expected improved quality of life (86 and 78%, respectively), as well as symptom relief with the treatment (83 and 87%, respectively), whereas more patients expected to experience than did oncologists (68 and 37%, respectively), see Table 2.

**Table 2:** Patients’ and oncologists’ NEOetic-EIT questionnaire results.

Variables	M (SD)	Unsure	Very low 0–25%	Low 26–50%	High 51–75%	Very high 76–100%
Patients’ expectations						
Expectations concerning cure	81 (31)	12	3	7	20	58
Expectations for improved quality of life	86 (25)	8	2	5	21	64
Expectations of relief of cancer symptoms	84 (26)	9	2	6	24	59
Expectation of experiencing many side effects	45 (36)	30	14	23	18	15
Doctors’ expectations						
Expectations concerning cure	44 (24)	5	41	34	15	6
Expectations for improved quality of life	83 (18)	–	–	14	37	49
Expectations of relief of cancer symptoms	84 (18)	–	1	13	35	52
Expectation of experiencing many side effects	46 (28)	9	29	39	11	12

M, Mean; SD, standard deviation.

### Factors affecting expectations in patient and oncologist treatment

There were no significant differences between male and female physicians in their assessment of the expectation concerning a cure, while there were differences regarding the rest of the expectations. More male versus female oncologists believed that antineoplastic treatment would lead to better patient outcomes (91.2% vs. 81.6%, respectively; *t* = 3.944, *p* = 0.001); relief of cancer symptoms (91.6% vs. 82.3%, respectively; *t* = 3.752, *p* = 0.001) and cause many side effects (53.4% vs. 44.7%, respectively; *t* = 2.264, *p* = 0.024). As for participants with advanced lung cancer, there were no differences between men and women in their treatment expectations.

Expectations for improved quality of life and relief of cancer symptoms negatively correlated with physicians’ age (*p* < 0.01) and years of experience (*p* < 0.01). Younger oncologists and those with fewer years of experience had higher expectations that the treatment would provide better quality of life and cancer symptom relief than older, more veteran oncologists (see Table 3). In patients with advanced lung cancer, treatment expectations were unrelated to patients’ age, physicians’ age, years of experience, or the doctor-patient relationship as assessed by the STAR-P questionnaire.

**Table 3:** Correlations between prognostic prediction and sociodemographic variables.

Variables	Doctors’ age	Years of experience	Patients’ age	STAR-C	STAR-P
Clinician expectations					
Expectations concerning cure	−0.053	−0.021	0.006	0.076	–
Expectations for improved quality of life	−0.168*	−0.183*	−0.043	0.057	–
Expectations for cancer symptom relief	−0.160*	−0.177*	0.002	0.069	–
Expectation of experiencing many side effects	0.041	0.022	−0.116	−0.027	
Patient expectations					
Expectations concerning cure	0.094	0.094	−0.087	–	−0.112
Expectations for improved quality of life	0.027	0.023	−0.038		−0.085
Expectations for cancer symptom relief	0.088	0.079	−0.039	–	−0.045
Expectation of experiencing many side effects	−0.012	−0.007	0.013	–	0.057

*p < 0.01.

### Data at 3 months of systemic cancer treatment

The most common side effects of any grade reported by participants after 3 months of treatment were fatigue (41.2%), loss of appetite (27.9%), peripheral neuropathy (27.4%), skin toxicity (27.2%), and pain (25.2%). After 3 months, 4% achieved a complete response; 58% displayed a partial response; 21% stabilized with systemic treatment, while 17% experienced progression, and 11% were deceased.

## Discussion

In this study, we explored expectations regarding treatment for unresectable, advanced lung cancer from the perspectives of both patients and medical oncologists. Our findings revealed that oncologists held greater expectations of positive outcomes in patients with a better baseline prognosis, such as those with ECOG 0, non-small cell lung cancer, unresectable locally advanced (as opposed to metastatic), newly diagnosed (versus recurrent), and biomarker-based treatment. In contrast, we found no distinction concerning patients’ treatment expectations according to sociodemographic or clinical factors. Participants tended to have high expectations about a cure, contrary to the clinicians’ lower expectations in this regard. Nevertheless, both anticipated similar improvements in quality of life. Although patients expected more side effects. Among oncologists, expectations varied by gender, with male oncologists having higher expectations of improvement and symptom relief than female oncologists, and these expectations decreased with age and experience. In patients, there were no differences in expectations based on gender, age, or doctor-patient relationship.

The patient sample displayed baseline demographic characteristics similar to those reported in earlier studies. Many of our subjects are elderly, with a mean age of 65.5 years, and 38% were female. These findings are consistent with the recent increase in lung cancer incidence among women (Siegel et al., 2020). As for marital status, a significant majority of our subjects are married or have partners (76.5%). Although marital status alone does not guarantee social support, it highlights the potential importance of family in the cancer patient’s experience. Family support plays a crucial role in managing the illness, and marital status has been identified as a potential independent predictor of survival in this population (Mystakidou et al., 1996; Chen et al., 2020). As for education, the proportion of the sample with a primary-level education (39.5%) resembles previous studies, indicating a diversity in educational levels among lung cancer patients (Özdemir et al., 2023). Notably, most patients were retired or unemployed (71.1%), which can be attributed to the higher average age and the severity and symptoms of unresectable, advanced lung cancer at the time of diagnosis. Future studies should explore the impact of a cancer diagnosis on these individuals’ work capabilities (Christalle et al., 2019). Histologically, most were non-small cell lung carcinomas (72.1%) and, of them, we observed a predominance of adenocarcinoma (40%). This is in line with trends reported in epidemiological studies indicating an increase in the proportion of adenocarcinomas compared to other histological subtypes (Travis et al., 2011).

All the medical oncologists who participated in our study were lung cancer subspecialists; most were young, with mean age of 36.2 years (range: 28–56 years), and female (65%). Limited data currently exist in the literature for comparison; however, these results are in line with current trends in oncology that exhibit more female professionals (Associatin of American Medical Colleges, 2020). The average experience of oncologists was 11.2 years, varying from 4 to 32 years. This reflects that professionals at different stages of their careers, participate in this study. This is relevant, inasmuch as pre-existing studies have identified that a physician’s experience impact decision-making and treatment expectations (Meropol et al., 2016). We also found this to be the case in our study, in that younger oncologists with fewer years of experience had higher expectations that the treatment could improve quality of life and control cancer symptoms compared to older or more veteran oncologists.

The analysis of patient expectations revealed a significant mismatch between their assumptions and the clinical reality of unresectable, advanced lung cancer. Despite the severity and advanced stage of the disease, most patients expressed high or very high expectations concerning being cure (58%) and having improved quality of life (85%). This discrepancy raises important considerations for clinical practice and effective communication between physicians and patients. The persistent hope for a cure among patients could be attributed to several reasons, such as not fully understanding the disease’s severity, using optimism as a coping strategy, or even the influence of hopeful messages from the oncologist, surroundings, or external sources. This underscores the need for careful and honest communication by healthcare professionals to align expectations with clinical reality.

The phenomenon of a disparity between patient expectations and clinical reality is not exclusive to lung cancer; rather, it is seen across several types of neoplasms. Existing literature regarding other cancers displays patterns in patient expectations, emphasizing its complexity in world of oncology. For instance, past studies in breast cancer have also identified this issue, with patients maintaining hope for a cure despite disease progression through different therapeutic lines (Hack et al., 2005). Nevertheless, a former study by our group found that women with breast cancer had more realistic prognostic awareness, though it was associated with greater psychological distress (Carmona-Bayonas et al., 2023). Similarly, the discrepancy between expectations and reality may reflect the influence of psychosocial factors on patient perception. The need for hope and optimism, especially in situations of severe illness, can lead patients to maintain high expectations for a cure. This has been documented in subjects with breast or prostate cancer (Rottmann et al., 2016; Wittmann et al., 2022) in whom anxiety, fear, and the need to find meaning in illness can contribute to maintaining high expectations concerning a cure. This may translate into a negative impact on the patient’s quality of life. Previous studies in ovarian and head and neck cancer have associated expectations unaligned with clinical reality with a decline in people’s quality of life (Wu et al., 2013; Lehto and Stein, 2009). From all the above, it becomes clear that the mismatch between patient expectations and clinical reality is a widespread phenomenon in oncology. To date, there is no established strategy to improve this situation.

Analysis of these data from the oncologist’s perspective reveals significant divergence *vis-a-vis* the patient’s viewpoint, stressing the challenges of communication in oncology. Correspondingly, there is a low expectation surrounding a cure among oncologists aware of the poor prognosis of advanced lung carcinoma. Prior studies have demonstrated the opposite, i.e., an optimistic tendency among healthcare professionals regarding oncological treatment in the context of advanced disease (Christakis and Lamont, 2000). Furthermore, previously published studies have determined that this optimism may be influenced by factors such as the overestimation of therapeutic abilities and resistance to accepting loss of control over the treatment outcome. In this respect, our study is innovative, as it is a monographic study on lung cancer with subspecialists in this neoplasm, principally from university hospitals with a high patient volume, which could lead to a more accurate adjustment of prognosis and treatment purpose.

Another important aspect relates to expectations of side effects. The lower expectation of side effects among oncologists compared to patients reflects the clinical perception of treatment tolerability. This is consistent with previous studies suggesting that healthcare professionals may underestimate the burden of side effects perceived by patients (Basch et al., 2006; Fromme et al., 2004). Moreover, patients may not be aware that newer biological treatments and immune checkpoint inhibitors are associated with less toxicity than traditional chemotherapy (Rosell et al., 2012; Reck et al., 2019).

The present study has several limitations that should be considered. First, the potential impact of other variables, such as coping mechanisms, spirituality, and socioeconomic status, on treatment expectations was not evaluated. Second, the approach the oncologist used to communicate information could have influenced the patient’s perception and expectations. Third, the type of healthcare system, communication practices, and therapeutic organization necessitate that these results be interpreted with caution when compared to those from other countries.

In conclusion, this study underscores the complexity of expectations held by both parties in the treatment of advanced lung cancer. While patients often have high expectations of being cured and enjoying improved quality of life, oncologists, with a better understanding of prognosis, tend to have more realistic expectations. This mismatch sheds light on the pivotal role of effective communication to align patient expectations with clinical reality. Furthermore, awareness must be raised surrounding awareness of the lower toxicity of targeted therapies and immune checkpoint inhibitors compared to traditional chemotherapy. This knowledge is vital for informed decision-making and proper management of patient expectations.

## Data Availability

The original contributions presented in the study are included in the article, further inquiries can be directed to the corresponding author.

## Footnotes

^1^www.neoetic.es
